# Statistical methods for identifying differentially expressed genes in RNA-Seq experiments

**DOI:** 10.1186/2045-3701-2-26

**Published:** 2012-07-31

**Authors:** Zhide Fang, Jeffrey Martin, Zhong Wang

**Affiliations:** 1 Biostatistics Program, School of Public Health, LSU Health Sciences Center, 2020 Gravier Street, 3rd floor, New Orleans, LA, 70112, USA; 2 Genomics Division, Lawrence Berkeley National Laboratory, Berkeley, CA, 94720, USA; 3 Department of Energy, Joint Genome Institute, Walnut Creek, CA, USA, 94598; 4 Staff Scientist, Group Lead for Genome Analysis, DOE Joint Genome Institute, 2800 Mitchell Dr., MS100, Walnut Creek, CA, 94598, USA

## Abstract

RNA sequencing (RNA-Seq) is rapidly replacing microarrays for profiling gene expression with much improved accuracy and sensitivity. One of the most common questions in a typical gene profiling experiment is how to identify a set of transcripts that are differentially expressed between different experimental conditions. Some of the statistical methods developed for microarray data analysis can be applied to RNA-Seq data with or without modifications. Recently several additional methods have been developed specifically for RNA-Seq data sets. This review attempts to give an in-depth review of these statistical methods, with the goal of providing a comprehensive guide when choosing appropriate metrics for RNA-Seq statistical analyses.

## Introduction

Transcriptomics holds the key to understanding how the information encoded in the genome is translated into cellular functions, and how this translation process responds to the changing environment. Given a transcriptome, or the collection of all the transcripts including both protein coding mRNAs and noncoding RNAs, one of the outstanding questions in transcriptomics is to accurately quantify the abundance of each transcript within different tissues and time points, and to correlate changes in abundance to genetic and environmental perturbation in order to understand genome function and adaptation.

Transcriptome profiling, or gene expression profiling, is the technology used to determine the steady state abundance of each transcript within a transcriptome. Transcriptome profiling is traditionally done using either quantitative RT-PCR (reviewed in [1]) to interrogate a few genes, or microarrays (cDNA array [2] or whole genome tiling array [3-5]) to investigate genome-wide transcriptional activity. Recently, as a result of the low cost of next generation sequencing technologies [6], transcriptome profiling by RNA sequencing (RNA-Seq) is becoming the method of choice because of its unprecedented sensitivity and accuracy (reviewed in [7,8]). Unlike prior technologies, next-generation sequencing technologies allow reference transcriptomes to be assembled directly from RNA-Seq data, thereby eliminating the need for existing reference genomes or transcriptomes [9]. This capability is particularly attractive for non-model organisms or microbial communities that lack high quality references.

**Figure 1:**
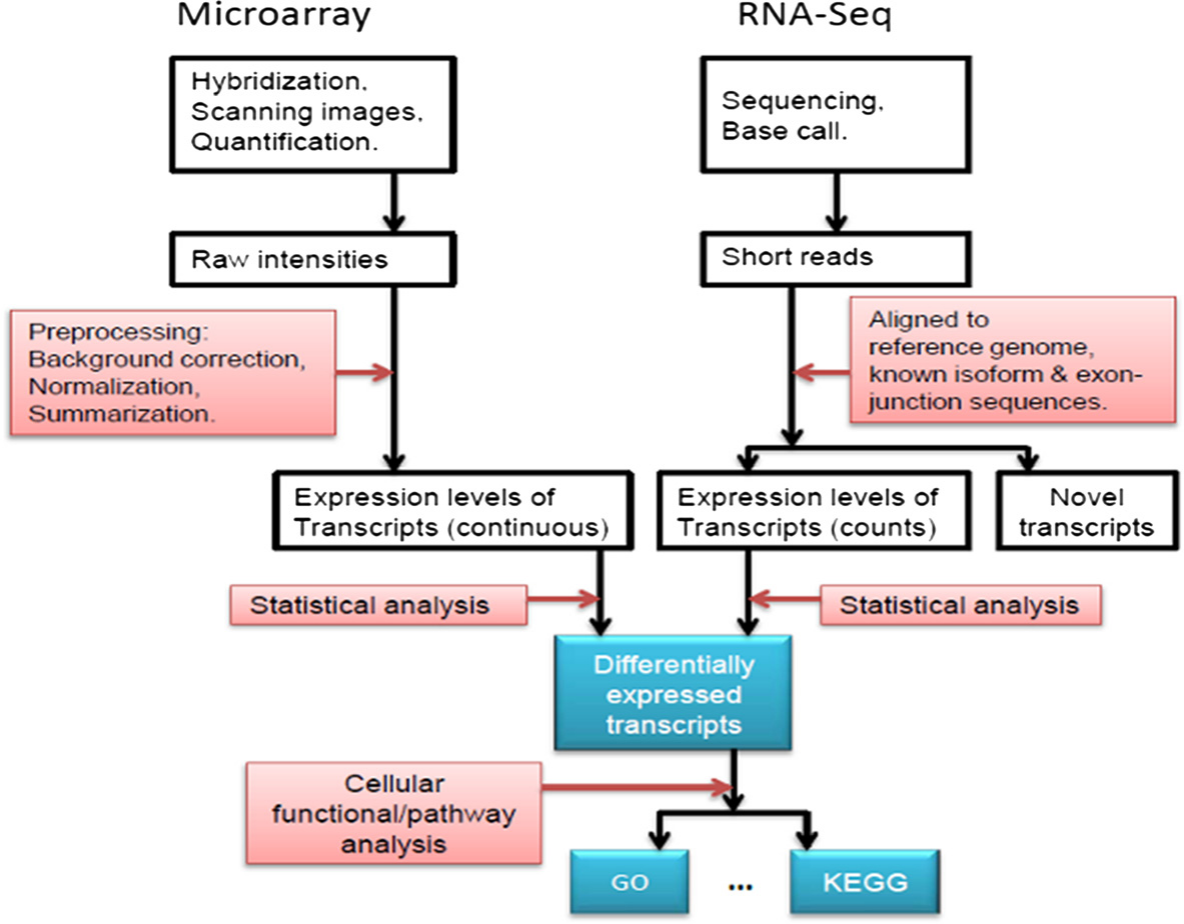
The analysis workflows of microarray and RNA-Seq data.

There are both shared and unique aspects in the experimental design and data generation phases of expression microarrays and RNA-Seq technologies, and these attributes are compared elsewhere [7,8,10,11]. For data analysis there are three major steps for both technologies: data preprocessing, statistical analysis and functional interpretation (Figure 1). Preprocessing microarray data normally includes background correction, normalization and summation, while preprocessing RNA-Seq data includes artifact filtering and short read alignment/assembly. The bioinformatics details involved in preprocessing microarray and RNA-Seq data have been reviewed previously [8,12,13]. After data preprocessing the expression level of each transcript is determined. For microarrays the levels are often represented as continuous numbers, while for RNA-Seq datasets expression levels are represented as discrete read counts. Statistical analysis is then performed to identify differentially expressed transcripts among different samples/conditions, and the results can be further analyzed to gain functional insights (Figure 1). In this review we focus on the statistical methods that are used to identify differentially expressed transcripts in RNA-Seq experiments. Some of them (for example, likelihood ratio test) have been used for microarray data analysis and then were adapted for RNA-Seq data analysis, while others were developed specifically for RNA-Seq.

Over the past decade, various statistical analysis tools have been developed to analyze expression profiling data generated by microarrays (Reviewed in [14]). Before these tools can be applied to RNA-Seq data, it is worth noting that microarray data and RNA-Seq data are inherently different. As mentioned earlier, microarray data is “analog” since expression levels are represented as continuous hybridization signal intensities. In contrast, RNA-Seq data is “digital”, representing expression levels as discrete counts. This inherent difference leads to the difference in the parametric statistical methods that are used since they often depend on the assumptions of the random mechanism that generates the data. For example, the normal distribution is a common distribution for statistical comparisons involving continuous data. It is generally assumed that the log intensities (or expression levels) in a microarray experiment are approximately normally distributed. In contrast, the Poisson, Binomial and Multinomial distributions are more suitable for modeling discrete data in an RNA-Seq experiment. Therefore a statistical method developed for microarray data analysis cannot be directly applied to RNA-Seq data analysis without first examining the underlying distributions. Recently several statistical methods have been developed to deal specifically with RNA-Seq count data [14-17]. In this review we summarize these methods, while focusing on the pros and cons of each method in the context of specific applications.

## RNA-Seq count data

As mentioned previously, the expression levels measured by RNA-Seq experiments are represented by the number of reads derived from each transcript in a transcriptome. We will not discuss the problem of resolving the expression levels of alternatively spliced transcript isoforms from a single gene, since this is still a challenge and undergoing active research [7,18-20]. Here, for simplicity, we use the term “gene” or “transcript” to generically refer to a spliced mRNA isoform, a non-coding RNA, a small RNA, or any product resulted from a transcriptional, splicing, or post-transcription-modification event.

RNA-Seq count data can be organized into a numerical (*p × n*) matrix (*M*), with *p* representing the number of genes and *n* the number of samples. We use phenotype to refer to an experimental condition, treatment, tissue, or time point. Typically the number of genes is far greater than the number of samples (*p* ≫ *n*). Another dimension often present in RNA-Seq datasets is the number of replicates. Since the RNA-Seq protocol is highly reproducible [21-23], technical replicates are usually not necessary, and instead 2–3 biological replicates are used to reduce the degree of noise resulting from biological variations. Generally we assume that there are *n*_*k*_ biological (or technical) replicates under the *k*^*th*^ phenotype, *k* = 1, 2, ⋯, *t*. As a result, a typical RNA-Seq data set has a series of two-dimensional sub-matrices containing non-negative integers (counts), with each sub-matrix being derived from a specific phenotype. Therefore, we have $n={\sum_{k=1}^{t}n}_{k}\text{,}$ and the data matrix can be arranged As M below, with the element *m*_*ij*_^(*k*)^ being the expression level of the *i*^*th*^ gene from the *j*^*th*^ replicate in the k^th^ phenotype, *i* = 1, 2, ⋯, *p*, *j* = 1, 2, ⋯, *n*_*k*_, *k* = 1, 2, ⋯, *t.*

(1) $$M=\left(\;\left[\begin{array}{cccc} m_{\;11}^{\left(1\right)} & m_{\;12}^{\left(1\right)} & \cdots & m_{\;1\;n_{\;1}}^{\left(1\right)}\\ m{\;}_{21}^{\left(1\right)} & m{\;}_{22}^{\left(1\right)} & \cdots & m{\;}_{2\;n_{\;1}}^{\left(1\right)}\\ m{\;}_{p1}^{\left(1\right)} & m{\;}_{p2}^{\left(1\right)} & \cdots & m{\;}_{p\;n_{\;1}}^{\left(1\right)} \end{array}\right]\;\left[\begin{array}{cccc} m_{\;11}^{\left(2\right)} & m_{\;12}^{\left(2\right)} & \cdots & m_{\;1\;n_{\;2}}^{\left(2\right)}\\ m{\;}_{21}^{\left(2\right)} & m{\;}_{22}^{\left(2\right)} & \cdots & m{\;}_{2\;n_{\;2}}^{\left(2\right)}\\ m{\;}_{p1}^{\left(2\right)} & m{\;}_{p2}^{\left(2\right)} & \cdots & m{\;}_{p\;n_{\;2}}^{\left(2\right)} \end{array}\right]\cdots \left[\begin{array}{cccc} m_{\;11}^{\left(t\right)} & m_{\;12}^{\left(t\right)} & \cdots & m_{\;1\;n_{\text{ t}}}^{\left(t\right)}\\ m{\;}_{21}^{\left(t\right)} & m{\;}_{22}^{\left(t\right)} & \cdots & m{\;}_{2\;n_{\text{ t}}}^{\left(t\right)}\\ m{\;}_{p1}^{\left(t\right)} & m{\;}_{p2}^{\left(t\right)} & \cdots & m{\;}_{p\;n_{\text{ t}}}^{\left(t\right)} \end{array}\right]\;\right)\;$$

## RNA-Seq count data normalization

An important consideration to make prior to statistical analysis is normalization. The sequencing depth, or library size, which is usually defined as the total number of aligned sequences in each sample, often varies from one sample to another. Denote *L*_*j*_^(*k*)^ as the sequencing depth for the *j*^*th*^ sample in the *k*^*th*^ phenotype. Then we have $L_{j}^{\left(k\right)}=\sum_{i\geq 1}m_{\mathrm{ij}}^{\left(k\right)}$. Normalizing count data transforms it from discrete to continuous. For example, the RPKM metric (Reads Per Kilobase of transcript per Million mapped reads [22]) is used to measure the relative expression level of a transcript. Although RPKM considers the length of the transcript, and thus allows for comparison among different transcripts, in most studies the gene length is not an issue because the comparison is made for the same gene between different conditions [24]. RPKM-based expression measurements cannot be directly used for the count-based models.

In this review we assume that the data in the matrix *M* are raw read counts without normalization. Unlike microarray data analysis which often requires sophisticated normalization procedures to compensate for biases introduced from sample loading, imaging, and other technical or biological factors [25], RNA-Seq data typically does not require a separate normalization step for two reasons: 1) The difference in the sequencing depths or library sizes between different samples is addressed through the parameterization of the underlying distributions (see below). 2) Some models already take into account the variation among biological replicates. For example, the over-dispersion parameter (discussed below) in the Negative Binomial model accounts for the variation across the biological replicates [26].

In the next section we will discuss the statistical methods that have been developed to address whether or not a gene is differentially expressed among a group of time points or conditions. In the case of t = 2, this problem reduces to the two-group (pair-wise) comparison.

## Statistical methods to detect differentially expressed genes

Several statistical methods have been proposed to detect the differentially expressed genes from a counts table (Table 1). The number of samples or replicates in a typical RNA-Seq experiment is usually small, thereby excluding the application of nonparametric methods that implement sample permutations. For this reason, here we focus on parametric methods only. These methods differ in their underlying data distributions, how they handle biological replicates, and their ability to perform multi-group comparisons. Some of these methods have been implemented in related *R/Bioconductor* packages (Table 1). Each of these methods is discussed in further detail below.

**Table 1:** A comparison of common statistical methods for RNA-Seq differential gene expression analysis

**Method**	**Underlying distribution**	**Recommended with biological replicates**	**Multi-group comparison**	**R/Bioconductor package**	**Reference**
Fisher’s exact Test	Poisson	No	No	No	[27]
Likelihood ratio test	Poisson	No	Yes	No	[21]
edgeR	Negative Binomial	Yes	Yes	Yes	[28]
DESeq	Negative Binomial	Yes	No	Yes	[15]
baySeq	Negative Binomial	Yes	Yes	Yes	[17]
BBSeq	Beta-Binomial	Yes	No	Yes	[29]
Two-stage poisson model	Poisson	Yes	Yes	No	[30]

## Methods based on the poisson distribution

In an RNA-Seq dataset, the expression level of a specific gene, *m*_*ij*_^(*k*)^, is defined as the total number of short sequences which aligned to the gene. That is, it is the sum of a series of random events. Each event corresponds to a short sequence and follows a Bernoulli distribution with the probability of success equating to the probability that the sequence aligns to the gene. Since the read alignments can be assumed to be independent, the distribution of *m*_*ij*_^(*k*)^ can be approximated by a Poisson distribution, *Poi*(*μ*_*i*_^(*k*)^), with *μ*_*i*_^(*k*)^ being the mean. This Poisson model is verified in the case where there are only technical replicates using a single source of RNA [21]. For the i^*th*^ gene, the statistical null hypothesis in testing different expression levels across phenotypes is that all of the means are equal. The statistical test procedures based upon Poisson modeling are reviewed in the next subsection.

### Fisher’s exact test

This method can be used for comparing two phenotypes (t = 2). For the i^*th*^ gene, we can form a 2х2 contingency table for its expression values in the matrix M [27], Table 2.

**Table 2:** The 2×2 contingency table for one gene

	**Gene**	**Not Gene**
**Phenotype 1**	*m*_*il*_^(1)^	*L*_*l*_^(1)^ − *m*_*il*_^(1)^
**Phenotype 2**	*m*_*il*_^(2)^	*L*_*l*_^(2)^ − *m*_*il*_^(2)^

The Fisher’s exact test is to test whether or not there exists a significant association between the gene expression and the phenotype, in other words, whether or not the odds ratio is significantly greater or less than 1. This test is based on the fact that with the assumption of Poisson sampling and fixed marginal totals, the count *m*_*i*1_^(1)^ follows a hyper-geometric distribution. The p value is the total of the hyper-geometric probabilities for outcomes at least as favorable to the alternative hypothesis (the gene expression in phenotype 1 is lower than that in phenotype 2 (the odds ratio is < 1), or vice versa) as the observed outcome [31]. A simple R function,

(2) $$\begin{array}{l} >x=matrix(cm{(}_{i1}^{\left(1\right)}\text{,}\;m_{i1}^{\left(2\right)}\text{,}\;L_{1}^{\left(1\right)}-m_{i1}^{\left(1\right)}\text{,}\;L_{1}^{\left(2\right)}-m_{i1}^{\left(2\right)})\text{,}\;nrow=2)\\ >fisher.test(x,alternative=c{(}^{\prime \prime }two.sided^{\prime \prime }{,}^{\prime \prime }greater^{\prime \prime }{,}^{\prime \prime }less^{\prime \prime })) \end{array}$$

will give the p value of the test.

Since the null hypothesis of independence in Fisher’s exact test is equivalent to the null hypothesis that the odds ratio is equal to 1, one can avoid a potential false positive due to the difference in the sequencing depths. As an example, consider the case: *m*_*i*1_^(1)^ = 10, *m*_*i*1_^(2)^ = 20, *L*_1_^(1)^ = 1*e* + 6, *L*_1_^(2)^ = 2*e* + 6. The estimated odds ratio is 1 (no association by Fisher’s exact test), but the fold change is 2 (possibly declared as differential by other tests based on fold change). Furthermore, though Fisher’s exact test was designed for analyzing datasets without replicates, if there are replicates and Poisson sampling holds true, the test can still be applied – one simply sums up the replicates under the same condition to form the 2×2 contingency table.

The p value obtained above is for a single gene. As in the analysis of expression data from microarray experiments, there are thousands of genes in one RNA-Seq experiment and thus we need to consider the problem of an inflated false positive rate due to multiple hypothesis testing. This problem can be addressed by directly adjusting p values or calculating q values [32,33]. Many methods have been proposed to calculate adjusted p-values, including Bonferroni’s single-step adjusted p-values, Holm’s step-down adjusted p values [34], Hochberg’s step-up adjusted p-values [35], and many more. The R function mt.raw2adjp() in the R/Bioconductor package multtest computes adjusted p values, with nine different computing procedures. Q values can be obtained by the R function qvalue() in the R/Bioconductor package qvalue. All of these functions take the vector of raw p values as the input argument.

### Likelihood ratio test

Marioni et al. assumed that the gene count *m*_*ij*_^(*k*)^ follows a Poisson distribution, *Poi*(*μ*_*i*_^(*k*)^ = *L*_*j*_^(*k*)^*ν*_*i*_^(*k*)^), where *ν*_*i*_^(*k*)^ represents the proportion of gene transcript copies of the ith gene in all samples under the kth phenotype (k = 1, 2, for pair-wise comparison), and then used the likelihood ratio test to identify the differentially expressed genes [21]. The purpose of incorporating the sequencing depth (*L*_*j*_^(*k*)^) parameter into the Poisson mean is to reduce the variation in sequencing depth. If we look into the significance of differential expressions of genes on a gene-by-gene basis, the likelihood function and maximum likelihood estimations are easy to obtain. For the two-sided alternative hypothesis, by applying simple algebraic operations we have the likelihood ratio test statistic,

(3) $$\Lambda_{i}=-2\left((\sum_{j}m_{\mathrm{ij}}^{\left(1\right)})\log\left(\frac{\sum_{j}m_{\mathrm{ij}}^{\left(1\right)}+\sum_{j}m_{\mathrm{ij}}^{\left(2\right)}}{\sum_{j}m_{\mathrm{ij}}^{\left(1\right)}}\frac{\sum_{j}L_{j}^{\left(1\right)}}{\sum_{j}L_{j}^{\left(1\right)}+\sum_{j}L_{j}^{\left(2\right)}}\right)+(\sum_{j}m_{\mathrm{ij}}^{\left(2\right)})\log\left(\frac{\sum_{j}m_{\mathrm{ij}}^{\left(1\right)}+\sum_{j}m_{\mathrm{ij}}^{\left(2\right)}}{\sum_{j}m_{\mathrm{ij}}^{\left(2\right)}}\frac{\sum_{j}L_{j}^{\left(2\right)}}{\sum_{j}L_{j}^{\left(1\right)}+\sum_{j}L_{j}^{\left(2\right)}}\right)\right)\text{,}$$

And the p value for an individual gene can be calculated as the right tailed probability of a Chi-squared distribution with 1 degree of freedom. For the one-sided alternative hypothesis, *v*_*i*_^(1)^ > *v*_*i*_^(2)^ the p value is half of the above right-tailed probability of the Chi-squared distribution if the unconstrained maximum likelihood estimates of *v*_*i*_^(1)^, *v*_*i*_^(2)^ satisfy: $({\hat{v}}_{i}^{\left(1\right)}=)\frac{\sum_{j}m_{\mathrm{ij}}^{\left(1\right)}}{\sum_{j}L_{j}^{\left(1\right)}}>\frac{\sum_{j}m_{\mathrm{ij}}^{\left(2\right)}}{\sum_{j}L_{j}^{\left(2\right)}}(={\hat{v}}_{i}^{\left(2\right)})\text{;}$ or 0.5 otherwise [36]. Adjusted p values or q values to control the false positive rate can be obtained by the methods described in the previous subsection.

Poisson modeling is an appropriate fit not only for sequencing data with technical replicates [21], but also for those with biological replicates, as long as the sample mean is close to the sample variance. However, the requirement that the variance is the same as the mean excludes the application of the Poisson model to RNA-Seq data, should over-dispersion (defined below) occur. The likelihood ratio test may give misleading results if the assumptions about the sampling distribution are violated.

## Models for over-dispersed count data

Given a sampling distribution, over-dispersion occurs if the observed variance is greater than the assumed variance. In the Poisson model, over-dispersion occurs if the sample variance is greater than the sample mean. There could be several sources that cause over-dispersion in RNA-Seq data, including the variability in biological replicates due to heterogeneity within a population of cells, possible correlation between gene expressions due to regulation, and other uncontrolled variations. The existence of over-dispersion in real data was observed in several previous studies [26,30]. Popular models to safeguard against over-dispersion include the negative binomial distribution, beta-binomial distribution or two-stage Poisson distribution, as discussed below.

### Negative binomial model

As mentioned above, when over-dispersion is observed across the samples, the gene counts cannot be estimated accurately by a simple Poisson model. One way to handle this problem is to apply a Bayesian method – allowing the Poisson mean to be a random variable and then model the gene counts by the marginal distribution of *m*_*ij*_^(*k*)^. Specifically, assume that the Poisson mean follows a Gamma distribution with the scale parameter *μ*_*i*_^(*k*)^*ϕ* and the shape parameter 1ϕ, then the marginal distribution of the gene count has a Negative Binomial distribution with mean *μ*_*i*_^(*k*)^ and the variance *μ*_*i*_^(*k*)^(1 + *μ*_*i*_^(*k*)^*ϕ*) [37]. The Negative Binomial distribution can model the over-dispersed Poisson gene count where *ϕ* > 0 and reduces to the Poisson distribution as *ϕ* → 0. The R/Bioconductor package “edgeR” applies this model to detect the differentially expressed genes in RNA-Seq data [28], where the mean of the Negative Binomial is rewritten as (*μ*_*i*_^(*k*)^ = *L*_*j*_^(*k*)^*ν*_*i*_^(*k*)^) to adjust for the difference in sequencing depths across the samples. The ith gene is differentially expressed if the parameters *v*_*i*_^(*k*)^ are significantly different across phenotypes. For simple, pair-wise comparisons between phenotypes, the Negative Binomial parameters are estimated by conditional maximum likelihood and quantile-adjusted conditional maximum likelihood [26,37], and then an exact test (similar to Fisher’s exact test) is carried out to generate p values for individual genes. These can be completed by using the R function “exactTest()” with options for different estimates of the dispersion. For complex, mutligroup comparisons among phenotypes, edgeR applies the Cox-Reid profile-adjusted likelihood method to estimate the Negative Binomial parameters [38], and then uses the generalized linear model likelihood ratio test (R functions glmFit() and glmLRT()) to discover differentially expressed genes.

While the relationship between the Negative Binomial mean and variance simplifies the estimation of these parameters, it may result in some variation unexplained by the model and thus potentially introduce selection biases in the differentially expressed genes. Anders and Huber extended the method in edgeR by hierarchically modeling this mean-variance relationship [15]. Their method is implemented in a R/Bioconductor package, called DESeq. The R function nbinomTest() or nbinomTestForMatrics() can return unadjusted p values for individual genes. The adjusted p values from the “BH” method [39] are also given by the first function. To our knowledge, DESeq is currently limited to pair-wise comparisons.

Another modification to edgeR is given by Hardcastle and Kelly [17]. Their method is based on an empirically Bayesian approach and can be used for multi-group comparisons as well as for pair-wise comparisons. The gene count is also modeled by the Negative Binomial distribution. For each gene, instead of calculating a p value, a posterior probability is obtained for each comparison (alternative) among phenotypes. The probability of differential expression of a gene is defined as the sum of the posterior probabilities for all possible comparisons. Then, the genes are ranked based upon the probability of differential expression This method is implemented in the R/Bioconductor package, baySeq. The R function getPosteriors() returns the posterior likelihoods of comparisons. One of the disadvantages of this method is that it is more computationally expensive since all types of comparisons (alternatives) are considered and the number of comparisons increases dramatically when the number of phenotypes increases.

### Beta-binomial model

Zhou, Xia and Wright model the gene count in a sample with a Beta-Binomial distribution [29]. Assuming that whether or not a short sequence is mapped to a particular transcript follows a Bernoulli law with a mapping probability θ, then θ has a prior of the Beta distribution. The introduction of randomness to the mapping probability is to account for the over-dispersion in the gene count data, with the over-dispersion being explained by a Beta distribution parameter φ. The maximum likelihood estimates of parameters (E(θ), φ) are obtained either by a free model in which both parameters are unrelated or by a constrained model in which a mean-overdispersion relationship is assumed. A logistic model is fitted to model the relationship between the mean E(θ) and the design matrix of covariates including the indicator variables for phenotypes. Then the significance of an indicator variable is determined by a Wald statistic (the ratio of the corresponding coefficient in the fitted logistic model and its standard error) and indicates whether or not the gene is differentially expressed. This method is implemented in the R package, BBSeq, which is available on this website (last accessed on 03/07/2012): http://www.bios.unc.edu/research/genomic_software/BBSeq/. Two R functions, *free.estimate() and constrained.estimate()*, can generate the raw p values for genes in pair-wise comparisons. However, no function in the package directly gives the p values for multi-group comparisons.

The Beta-Binomial model has been widely used to model the over-dispersed, discrete count data. For example, it was applied to analyze tag count data derived from the Serial Analysis of Gene Expression (SAGE) [40]; tag count data obtained from Digital Gene Expression profiling [41]; and spectral count data generated from label-free tandem mass spectrometry-based proteomic experiments [42]. To model RNA-Seq data with a Beta-Binomial distribution, the probability that a short sequence is mapped to a specific transcript is implicitly assumed to be constant for all short sequences in a sample. We have not seen any verification or justification of this assumption in the literature.

### Two-stage poisson model

Auer and Doerge [30] proposed a Two-Stage Poisson Model for RNA-Seq data analysis, based upon the argument that the over-dispersion is most likely caused by within group variation in expression if the experiment includes independent biological replicates without a significant population structure. The method consists of two steps. In the first step, genes are classified into two exclusive classes, genes with or without over-dispersion, by using an adjusted score test on the hypothesis of whether or not the over-dispersion is present within the count data of a gene. Then in the second step, for genes without significant over-dispersion, differential expression is tested by a standard likelihood approach with maximum likelihood estimates being obtained under the Poisson model. Raw p values are calculated by an approximated Chi-squared distribution of degree one. For genes with significant over-dispersion, they use the quasi-likelihood statistic that is defined as the ratio of the likelihood statistic and an estimate of over-dispersion. Raw p values are calculated from an *F*-distribution. The built-in R functions "*glm()*" and "*deviance()*" can be used to obtain the likelihood ratio statistics. The detailed R code for p values can be downloaded from the author’s website (http://www.stat.purdue.edu/~doerge/software/TSPM.R), last accessed on 03/07/2012.

Under the model assumptions, the authors demonstrated that the Two-Stage Poisson Model is a powerful tool for detecting differentially expressed genes. However, if other confounding factors exist such that the levels of transcription within a phenotype are not stable, this method may not control the false positive rate well. Furthermore, as pointed out by the authors, this method requires a relatively higher degree of freedom (the difference between the sample size and the number of phenotypes) in order to be effective.

## Conclusion and future perspectives

Next-generation sequencing technologies are revolutionizing genomic/proteomic studies, providing high-throughput datasets with unprecedented precision and accuracy. The technology for profiling gene expressions and composition (RNA-Seq) has been widely applied in biological/medical research. Appropriate and powerful statistical analysis using RNA-Seq data is essential to the research.

**Figure 2:**
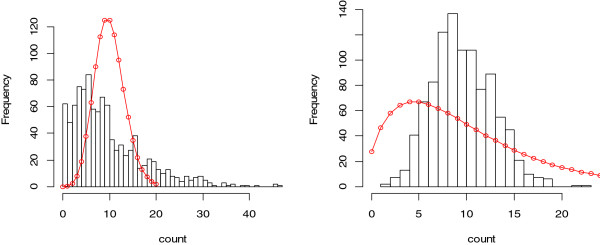
Histograms and wrongly fitted models for 1000 simulated data points.

Generating an accurate list of differentially expressed genes is the basis for pathway or gene set enrichment analysis. A gene set with a large number of false positives will compromise these analyses. The parametric approaches discussed here are preferable to nonparametric ones in order to increase the power of detection. However, the false positive rate may be dramatically increased if the assumptions of the model distribution are violated. In Figure 2, we demonstrate the difference between the histogram of 1000 data points simulated from an underlying distribution and the probability mass of an incorrectly fitted model (red curves). The data in the left panel are generated from a Negative Binomial distribution with mean 10 and over-dispersion 0.5 using the *R* function *rnegbin()*, but are modeled by a Poisson distribution with the probability mass:

(4) $$p(k)=\frac{\lambda^{k}e^{-\lambda }}{k!}\text{,}\;k=0\text{,}\;1\text{,}\;2\text{,}\cdots \cdots \text{,}$$

where the mean *λ* = 10. Those in the right panel are generated from a Poisson distribution with mean 10 using the *R* function *rpois()*, but are modeled by a Negative Binomial distribution with the probability mass:

(5) $$p(k)=\frac{\Gamma (k+\phi^{-1})}{\Gamma (k+1)\Gamma (\phi^{-1})}{\left(\frac{1}{1+\mu \phi }\right)}^{\frac{1}{\phi }}{\left(\frac{\mu }{\mu +\phi^{-1}}\right)}^{k}\text{,}\;k=0\text{,}\;1\text{,}\;2\text{,}\cdots \cdots \text{,}$$

where *Γ* represents the gamma function and the mean *μ* = 10, the over-dispersion parameter *ϕ* = 0.5. The values for the small circles in the fitted models are calculated as the product of 1000 and *p(k).* The differences in both cases are not negligible, indicating the seriousness of wrong assumptions about the model.

To our knowledge, there is no paper in the literature which studies the efficacy of these methods when the model assumptions do not hold. Given the limitation of small sample sizes in RNA-Seq experiments, robust test procedures which safeguard against the departure of model assumptions are necessary.

Most of the proposed methods produce raw p values for genes based upon the approximate/asymptotic null distribution. This approximation performs well for highly expressed genes but performs poorly for lowly expressed genes. This may create bias during the selection of differentially expressed genes. Some authors simply filter out lowly expressed genes. This is very subjective, and some important genes, which are expressed in one condition but not in another, may be excluded from the result. New testing approaches, which are powerful and effective for both highly and lowly expressed genes, are still needed.

## Competing interests

The authors declare no competing interests.

## Authors’ contributions

ZF, JM and ZW wrote the manuscript. All authors read and approved the final manuscript.
